# Assessment of *MYC*/PTEN Status by Gene-Protein Assay in Grade Group 2 Prostate Biopsies

**DOI:** 10.1016/j.jmoldx.2021.05.006

**Published:** 2021-08

**Authors:** Daniela C. Salles, Thiago Vidotto, Farzana A. Faisal, Jeffrey J. Tosoian, Liana B. Guedes, Andrea Muranyi, Isaac Bai, Shalini Singh, Dongyao Yan, Kandavel Shanmugam, Tamara L. Lotan

**Affiliations:** ∗ Department of Pathology, Johns Hopkins University School of Medicine, Baltimore, Maryland; † Department of Urology, Johns Hopkins University School of Medicine, Baltimore, Maryland; ‡ Department of Urology, University of Michigan, Ann Arbor, Michigan; § Roche Tissue Diagnostics, Tucson, Arizona; ¶ Department of Oncology, Johns Hopkins University School of Medicine, Baltimore, Maryland

## Abstract

This study leveraged a gene-protein assay to assess *MYC* and PTEN status at prostate cancer biopsy and examined the association with adverse outcomes after surgery. *MYC* gain and PTEN loss were simultaneously assessed by chromogenic *in situ* hybridization and immunohistochemistry, respectively, using 277 Grade Group 2 needle biopsies that were followed by prostatectomy. The maximal size of cribriform Gleason pattern 4 carcinoma (CRIB), the presence of intraductal carcinoma (IDC), and percentage of Gleason pattern 4 carcinoma at biopsy were also annotated. *MYC* gain or PTEN loss was present in 19% and 18% of biopsies, respectively, whereas both alterations were present in 9% of biopsies. Tumors with one or both alterations were significantly more likely to have non–organ-confined disease (NOCD) at radical prostatectomy. In logistic regression models, including clinical stage, tumor volume on biopsy, and presence of CRIB/IDC, cases with *MYC* gain and PTEN loss remained at higher risk for NOCD (odds ratio, 6.23; 95% CI, 1.74–24.55; *P* = 0.005). The area under the curve for a baseline model using CAPRA variables (age, prostate-specific antigen, percentage of core involvement, clinical stage) was increased from 0.68 to 0.69 with inclusion of CRIB/IDC status and to 0.75 with *MYC*/PTEN status. Dual *MYC*/PTEN status can be assessed in a single slide and is independently associated with increased risk of NOCD for Grade Group 2 biopsies.

Patients with Grade Group 2 (Gleason score 3 + 4 = 7) prostate cancer on needle biopsy have a broad range of clinical outcomes. Although most Grade Group 2 cases receive definitive treatment with radical prostatectomy or radiation, some active surveillance programs enroll patients with favorable risk Grade Group 2 disease.^1^ Standard clinicopathologic parameters, including prostate-specific antigen (PSA) and metrics of tumor volume at biopsy, are of limited utility in predicting outcomes for Grade Group 2 disease, and additional biomarkers are needed. Recent refinements of pathologic evaluation of prostate cancer, including identification of intraductal carcinoma, have improved our ability to identify aggressive tumors.^2^^,^^3^ Another morphologic factor that emerged in recent studies is the presence of cribriform Gleason pattern 4 disease because it is associated with up-staging and worse prognosis after prostatectomy.^3^, ^4^, ^5^, ^6^ In addition, a number of genomic, largely RNA-based biomarkers have been marketed for use in patients with intermediate-risk prostate cancer.^7^^,^^8^ Although many of these add to clinicopathologic parameters for the prediction of adverse outcomes, they require considerable amounts of tissue (often 5 to 10 unstained slides from a biopsy block), are highly dependent on adequate RNA preservation (which can be unpredictable in formalin-fixed, paraffin-embedded [FFPE] tissue), and cost on the order of $3000 to perform. Most notably, these markers generally lack studies that evaluate cost-benefit ratios, and most are only available in the United States.^9^

Apart from RNA signatures, a number of DNA-based alterations are common in primary prostate cancer and reproducibly associated with aggressive disease. Our group has previously found that *PTEN* deletions can be sensitively detected using a simple immunohistochemistry (IHC) assay and are significantly associated with adverse oncologic outcomes in surgically treated primary prostate cancer, including up-grading and up-staging at prostatectomy, biochemical recurrence, and metastasis.^10^, ^11^, ^12^, ^13^ Other work has found that copy number alterations at 8q24, including *MYC* gene gain, may be associated with Grade Group upgrade at prostatectomy.^14^^,^^15^ During the last few years, automated methods for brightfield (chromogenic) DNA *in situ* hybridization have been optimized, and these techniques can be performed in a dual-plex fashion with immunohistochemical staining, known as a gene-protein assay.^16^ We tested a clinical grade gene-protein assay to assess *MYC* and PTEN status simultaneously on a single biopsy slide. We hypothesized that the addition of these two molecular biomarkers to contemporary clinicopathologic parameters would improve the ability to predict which tumors have adverse pathology at radical prostatectomy.

## Materials and Methods

### Patients and Tissue Samples

Samples used in this study were previously described in a study examining the utility of a single-plex assay for PTEN in the setting of Grade Group 2 prostate cancer at biopsy.^17^ Briefly, with institutional review board approval, the Johns Hopkins Pathology database was queried for all needle biopsies performed between 2000 to 2014 at the Johns Hopkins Hospital (JHH) containing a maximum of Grade Group 2 cancer and followed by a radical prostatectomy at JHH. Where available, all Grade Group 2 biopsies were re-reviewed for this study, and grading was performed based on the International Society of Urological Pathology (ISUP) 2014 updated system. For each biopsy, the percentage of tumor involving the core was visually estimated by the reporting pathologist as described previously.^18^ The proportion of cores involved by tumor was retrospectively calculated by dividing the number of involved cores by the total number of reported cores submitted for the case. All the biopsies were followed by radical prostatectomy at JHH, and all the radical prostatectomy tissue was entirely submitted for histologic evaluation. The grading for the radical prostatectomies was performed as defined by the 2005 ISUP Consensus Conference on Gleason Grading of Prostatic Carcinoma.^19^ At JHH, men were followed up with PSA assays every 3 months after surgery for the first year, semiannually for the second year, and annually thereafter. A detectable serum PSA level of at least 0.2 ng/mL was evidence of biochemical recurrence.

To not exhaust all tumor tissue from the case, a single index biopsy block from each case containing the maximum percentage of core involvement by Grade Group 2 tumor was selected for further morphologic (cribriform Gleason pattern 4 carcinoma, intraductal carcinoma, and percentage of Gleason pattern 4 carcinoma) and immunohistochemical (p63) studies described below. This was the only block evaluated for cribriform or intraductal carcinoma; if present on another block, this was not included in further analysis. This same block was used for the retrospective *MYC*/PTEN gene-protein assay, and this assay was scored in 50 randomly selected evaluable tumor nuclei within the biopsy as described below. In most cases, the single block selected for study comprised two biopsy cores from the same anatomical location. An additional group of 28 biopsies from 2018 to 2019 with Grade Group 2 cancer were collected to compare the results of the *MYC*-PTEN gene-protein assay with two different anti-PTEN antibodies (see below).

### Intraductal Carcinoma, Cribriform Gleason Pattern 4 Carcinoma, and Percentage of Gleason Pattern 4 Scoring

**Supplemental Figure S1:**
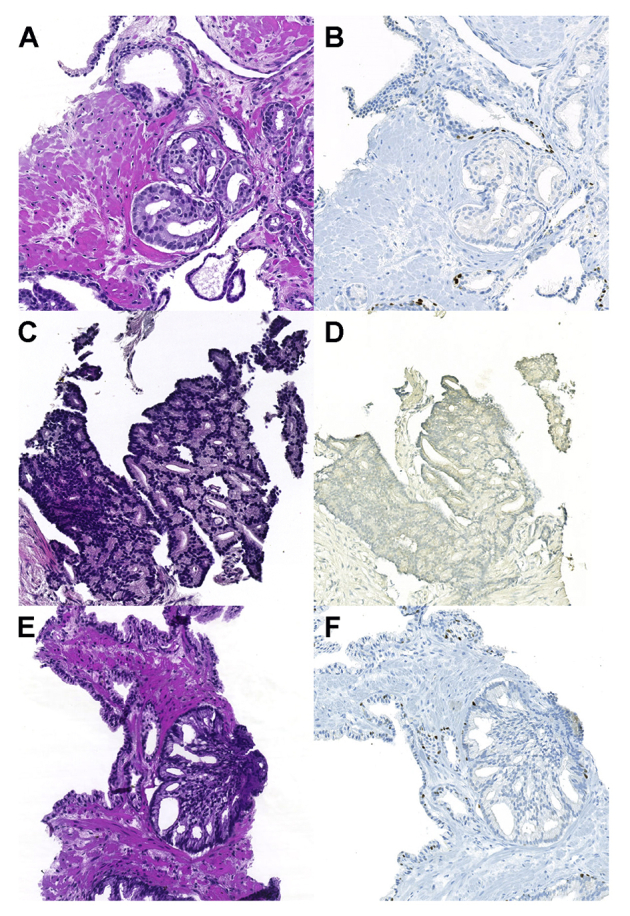
Representative cribriform and intraductal carcinoma. **A** and **B:** Invasive cribriform Gleason pattern 4 carcinoma with small (<200 um) maximal diameter: hematoxylin and eosin image (**A**) and p63 immunohistochemistry showing a lack of basal cells in cribriform foci, confirming invasive cancer **(B)**. **C** and **D:** Invasive cribriform Gleason pattern 4 carcinoma with large (>200 um) maximal diameter: hematoxylin and eosin image (**C**) and p63 immunohistochemistry showing lack of basal cells in cribriform foci, confirming invasive cancer **(D)**. **E** and **F:** lntraductal spread of prostate carcinoma: hematoxylin and eosin image (**E**) and p63 immunohistochemistry showing patchy basal cells around cribriform foci, confirming intraductal spread **(F)**. Original magnification, ×200.

Biopsy specimens with any size cribriform lesion in the index tumor block examined for *MYC*/PTEN analysis (see below) were subjected to p63 immunostaining to differentiate intraductal carcinoma from invasive cribriform Gleason pattern 4 carcinoma. p63 protein expression was detected using a mouse anti-p63 monoclonal antibody (clone 4A4, Abcam, Cambridge, UK). Staining was performed on a Discovery ULTRA automated staining platform. Presence of any p63-positive basal cells around a cribriform lesion allowed classification of that lesion as intraductal carcinoma. All lesions without basal cells were classified as invasive cribriform Gleason pattern 4 carcinoma and scanned hematoxylin and eosin images (Hamamatsu, Shizuoka, Japan) were used to digitally measure the maximal diameter of the largest cribriform Gleason pattern 4 focus present on the examined tumor block (Supplemental Figure S1). The percentage of Gleason pattern 4 carcinoma was determined for each case on the index block using visual estimation by a fellowship trained urologic pathologist (D.C.S.).

A total of 213 cases lacked large (maximal diameter >200 μm) cribriform Gleason pattern 4 carcinoma and intraductal carcinoma on initial analysis of the single index block. Of these, 139 (65%) had all Grade Group 2 cores examined in the initial index block described above and were likely to have no intraductal or cribriform Gleason pattern 4 carcinoma. There were 74 cases with additional Grade Group 2 tumor containing cores that were not included in the single block studied and thus may have had intraductal or cribriform Gleason pattern 4 carcinoma missed. From this group with unexamined Grade Group 2 cancer, 91 additional Grade Group 2 blocks were retrieved on 73 cases to assess for the presence or absence of intraductal carcinoma and cribriform Gleason pattern 4 carcinoma in these additional cores as described above.

### *MYC*-PTEN Chromogenic Gene-Protein Assay

The unstained slides used for the assay were cut in 2016 and stored at −20°C until the assay was performed in 2020. This assay has been described elsewhere for HER2/Neu.^16^ The gene-protein assay was performed on the BenchMark ULTRA automated staining platform. PTEN protein expression was detected using an anti-PTEN rabbit monoclonal antibody [clone D4.3 XP, Cell Signaling Technology (CST), Danvers, MA] and the OptiView DAB IHC Detection Kit (Ventana Medical Systems, Inc., Oro Valley, AZ). *MYC* gene and chromosome 8 status were assessed using MYC DNP Probe and Chromosome 8 DIG Probe with the *ultra*View SISH DNP Detection Kit and *ultra*View Red ISH DIG Detection Kit, respectively (Ventana Medical Systems, Inc.).

Briefly, FFPE tissue samples (4 μm) were deparaffinized and heat-pretreated with Cell Conditioning 1 for antigen retrieval (64 minutes). Specimens were incubated with the anti-PTEN (D4.3) rabbit monoclonal antibody for 16 minutes at 37°C, and the immunolocalized protein was visualized using the OptiView DAB IHC Detection Kit. After detection of PTEN protein, the tissue specimens were heat-pretreated with Cell Conditioning 2, followed by protease treatment with ISH Protease 2 for 16 minutes at 37°C. The samples were then denatured for 4 minutes at 80°C and hybridized for 6 hours at 46°C with MYC DNP Probe and Chromosome 8 DIG Probe Cocktail. The *MYC* and chromosome 8 signals were visualized using the Ventana *ultra*View SISH DNP Detection Kit and *ultra*View Red ISH DIG Detection Kit, respectively. All slides were counterstained with Hematoxylin II and Bluing Reagents for 4 minutes each, and coverslips were applied.

### *MYC*-PTEN Gene-Protein Assay Scoring

**Figure 1:**
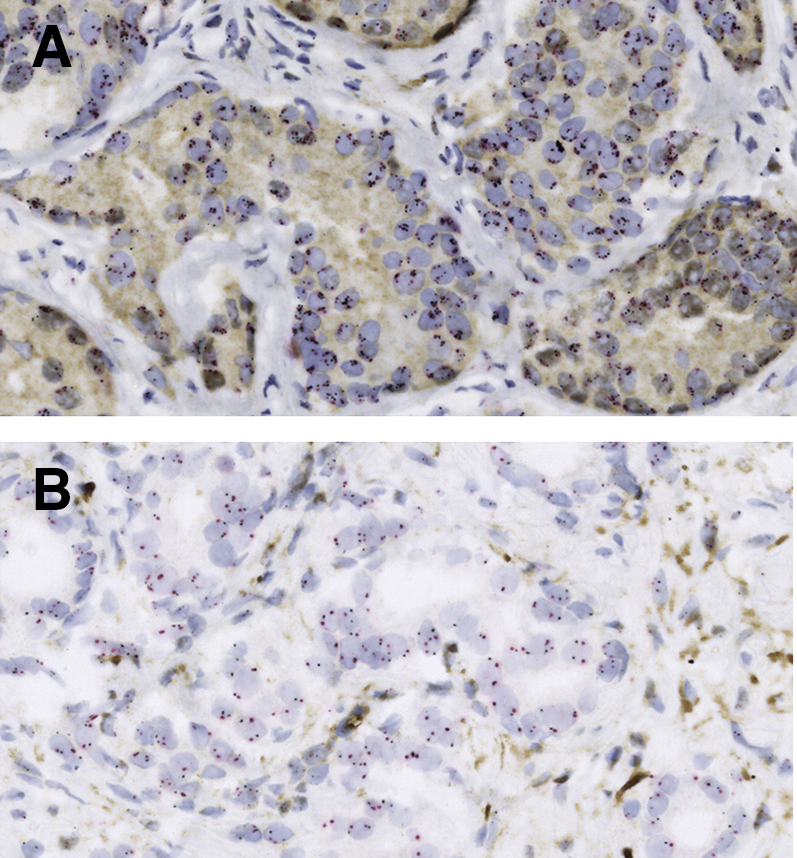
Representative *MYC*/PTEN gene-protein assay results in prostate biopsies. **A:** Representative case with high copy gain of *MYC* (>20% of cells with three or more copies; silver probe) and intact PTEN protein (brown). **B:** Representative case with intact *MYC* (<20% of cells with three or more copies; silver probe) and loss of PTEN protein (brown). Original magnification, ×400.

The PTEN IHC assay was blindly scored by two pathologists (S.S. and D.C.S. or T.L.) using a validated scoring system.^10^^,^^11^^,^^17^^,^^20^ In brief, a tumor biopsy specimen was considered to have homogeneous PTEN protein loss if the intensity of cytoplasmic and nuclear staining for PTEN was markedly decreased or entirely negative in all sampled tumor cells compared with surrounding benign glands and/or stroma (Figure 1). If PTEN was lost in some but not all tumor cells sampled in a given core, the core was annotated as showing heterogeneous PTEN loss. For the purposes of analysis, cases with heterogeneous PTEN loss and homogeneous PTEN loss were grouped together as PTEN loss cases. Some cases were scored as having ambiguous PTEN IHC results when the intensity of the tumor cell staining was light or absent in the absence of evaluable benign glands or stroma.

*MYC* gene status was assessed by enumeration of the *MYC* gene and chromosome 8 signals counted in 50 nuclei of cells for each case (Figure 1). Cases were scored as having inevaluable *MYC* status because of weak or lack of *in situ* hybridization signals related to preanalytical differences in sample preparation (time to fixation, type of fixative, underfixation or overfixation, and tissue thickness) and/or difficulties with signal enumeration because of nuclear PTEN staining. Previous studies have used variable cut-offs to define the presence or absence of the *MYC* gene gain. In a study performed by our colleagues in Gleason pattern 3 prostate cancer, any case with >30% of cells showing three or more copies of *MYC* was designated as *MYC* gain.^14^ However, previous *MYC* chromogenic *in situ* hybridization studies in B-cell lymphoma have defined a case as having *MYC* gain when there were >10% excess *MYC* signals or a mean of >2.2 *MYC* signals per nucleus.^21^ When compared with the Trock et al^14^ *MYC* fluorescence *in situ* hybridization study in prostate cancer, this cutoff was found to be exactly equivalent to a cutoff of ≥20% cells with three or more *MYC* copies. Furthermore, on receiver operating characteristic (ROC) analysis, the 20% cutoff indicated the maximal association with non–organ-confined disease in our cohort; thus, this cutoff was used in the current study to define *MYC* gain.

### Comparison of PTEN Scores from a Gene-Protein Assay to a Single-Plex IHC Assay

Previously published PTEN status from the single-plex IHC assay using the same primary antibody^17^ was compared with the scores generated from the gene-protein assay (performed on a deeper level after recutting the FFPE blocks). In total, 232 cases were available for comparison with a Cohen's κ value of 0.61 for all three categories (PTEN intact, heterogeneous PTEN loss, and homogeneous PTEN loss) or 0.66 for two categories [PTEN intact or PTEN loss (heterogeneous and homogeneous)] (Supplemental Table S1).

### Comparison of PTEN and *MYC* Scores from Different Gene-Protein Assays

To determine whether other anti-PTEN antibody clones performed similarly to the CST D4.3 antibody in the *MYC*-PTEN gene-protein assay, the Ventana PTEN (SP218) clone was additionally tested on a subset of the main Grade Group 2 cohort using previously cut and stored unstained slides as used for the main study. However, the intensity of PTEN immunostaining in the gene-protein assay was uniformly lower using the Ventana PTEN (SP218) clone compared with the CST D4.3 clone, making interpretation of PTEN status difficult. To determine whether this was attributable to the long-term storage of the unstained slides and the older FFPE block age, the *MYC*-PTEN assay was retested using both antibody clones on freshly cut slides from a separate group of 28 cases with Grade Group 2 prostate cancer on biopsy diagnosed from 2018 to 2019. The intensity of the PTEN stain increased on this newer and freshly cut cohort for both antibody clones to the extent that strong nuclear PTEN staining for the CST D4.3 clone at the 1:100 dilution obscured the interpretation of *MYC* status in many cases. The CST D4.3 clone was further tested at 1:200 dilution to resolve this issue. On blinded scoring of the CST D4.3 1:200 and Ventana PTEN (SP218) conditions in the gene-protein assay, 22 of the 22 interpretable cases had concordant *MYC* status using the 20% threshold for *MYC* gain. For PTEN status, there was agreement across the CST D4.3 1:100, CST D4.3 1:200, and Ventana PTEN (SP218) conditions for 24 of the 27 interpretable cases. For the remaining 3 cases, the SP218 clone had small regions with ambiguous staining in tumor glands (because of the focal lack of internal control staining), consistent with the overall less intense PTEN staining seen with this clone.

### Statistical Analysis

All statistical analyses were conducted with R software version 4.0.1 (R Foundation for Statistical Computing, Vienna, Austria). Clinical continuous variables, such as age, PSA levels, percentage of tumor involvement, and fraction cores involved, were compared using the Kruskal-Wallis test (for variables with three or more groups) or Wilcoxon test (for variables with two groups). Categorical variables were compared using the χ^2^ test. For analysis, *MYC* and PTEN status was combined into a four-possibility categorical variable.

**Supplemental Figure S2:**
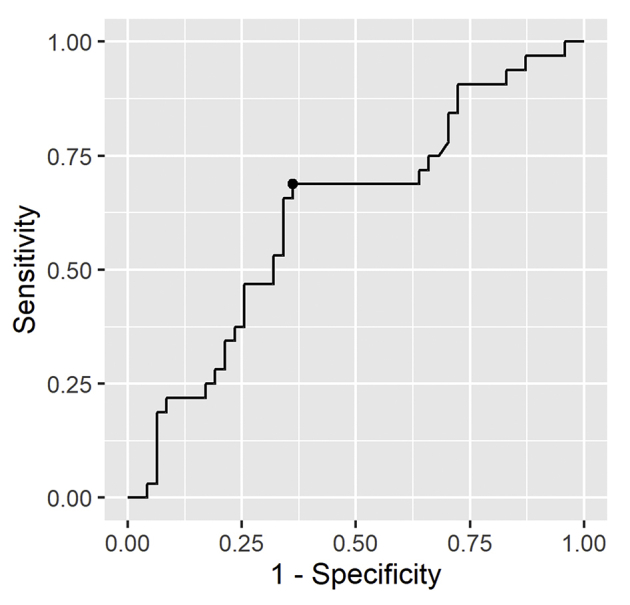
Receiver operator characteristic curve for association of maximal cribriform Gleason pattern 4 carcinoma diameter at biopsy with non–organ-confined disease at radical prostatectomy.

Because the maximal size of invasive cribriform Gleason pattern 4 carcinoma was measured as a continuous variable in all tumor samples, it was explored whether a cutoff point could be determined for the lesion size to facilitate measurement in the clinical setting. ROC analysis was therefore used for association with non–organ-confined disease to identify a cutoff with maximum sensitivity and specificity. The area under the curve (AUC) was calculated for the continuous cribriform Gleason pattern 4 lesion size and a maximal diameter of 200 μm was found to be the optimal cut point with respect to sensitivity and specificity for non–organ-confined disease. These analyses were performed using the *pROC* package in R software version 4.0.1 (Supplemental Figure S2).

Univariable and multivariable logistic regression models were used to calculate the odds ratios (ORs) and CIs for the occurrence of non–organ-confined disease. Univariable and multivariable models were also used to calculate the association between molecular changes (eg, *MYC* gain and PTEN loss) and cribriform Gleason pattern 4 lesions that were larger than the previously calculated size threshold of 200 μm (see previous paragraph and Supplemental Figure S2). The same models were generated with intraductal lesions as an endpoint. Multivariable models were generated with variables that had *P* < 0.05 in the univariable models.

Kaplan-Meier curves and log-rank tests were conducted to determine the association between biochemical recurrence-free survival and molecular changes, such as *MYC* gain and PTEN loss. Similar analyses were conducted using morphologic features of tumors, namely, intraductal carcinoma and cribriform Gleason pattern 4 lesions. These analyses were performed with the package *survival* in R software.

To identify changes in sensitivity and specificity after incorporating morphologic and molecular markers to classic clinicopathologic ROC curves, clinical, pathologic, and molecular variables were combined in multivariable logistic regression models. The output of these models was used to generate multiple AUCs. The first baseline AUC was generated using only variables used to calculate CAPRA (age, PSA, percentage of core involvement, and clinical stage) scores. Gleason scores at biopsy were not included because the investigated cohort was exclusively composed of 3 + 4 = 7 prostate tumors. This analysis was performed identically to that described previously.^17^

Next the presence of cribriform Gleason pattern 4 lesions and intraductal carcinoma and percentage of Gleason pattern 4 carcinoma were incorporated into the models to identify whether there was a significant increase in the AUC values. The effect of independently adding PTEN and *MYC* in the models and by combining all variables together (CAPRA, intraductal carcinoma, cribriform Gleason pattern 4 lesion, PTEN status, and *MYC* status) was also tested. Similar models were generated using CAPRA scores ranging from 0 to 10, which were generated after the dichotomization of age, PSA, percentage of core involvement, and clinical stage variables.

## Results

### Clinicopathologic and Molecular Features of the Cohort

Clinicopathologic features of the cohort of biopsy specimens included in this study are given in Table 1. Defined by p63 immunostaining, 11% of the biopsy specimens had intraductal spread of carcinoma present, whereas 19% had foci of large cribriform Gleason pattern 4 carcinoma present (maximal diameter >200 μm, see Materials and Methods for this cut-point selection) (Supplemental Figure S2). Large cribriform and intraductal carcinoma were significantly associated with one another (*P* < 0.0001), and their overlap is detailed in Supplemental Table S2. A total of 74 cases lacked intraductal or large cribriform Gleason pattern 4 carcinoma on the examination of the index tumor block and had additional tumor blocks with Grade Group 2 prostate cancer. Of these, 73 cases had additional blocks available for analysis, and 15 had large cribriform (maximal diameter >200 μm) (*n* = 13) or cribriform and intraductal foci identified (*n* = 2), comprising an additional 5% of the cohort.

**Table 1:** Clinical, Pathologic, and Molecular Features of the Investigated Cohort

Clinical feature	Finding (*N* = 277)
Preoperative parameters	
Age, median (range), years	63 (41–75)
Self-reported African American race, *n* (%)	47 (18)
PSA, median (range)	5.3 (1.3–33.8)
Clinical stage, *n* (%)	
T1c	194 (73)
T2a	33 (12.4)
≥T2b	39 (14.6)
Tumor involvement, median (range), %	70 (5–100)
Fraction cores involved, median (range), *n*	0.30 (0.05–1)
Bilateral disease, *n* (%)	148 (53)
Intraductal carcinoma, *n* (%)	31 (11)
Cribriform Gleason pattern 4 (≤200 μm), *n* (%)	77 (28)
Cribriform Gleason pattern 4 (>200 μm) *n* (%)	55 (19)
Gleason pattern 4, median (range), %	10 (0–90)
Postoperative parameters	
Grade Group at RP, *n* (%)	
1 (Gleason score 6)	78 (28.2)
2 (Gleason score 3 + 4 = 7)	148 (53.6)
3 (Gleason score 4 + 3 = 7)	38 (13.7)
4 (Gleason score 8)	6 (2.1)
5 (Gleason score 9–10)	6 (2.1)
Extraprostatic extension, *n* (%)	76 (27.4)
Seminal vesicle involvement, *n* (%)	10 (3.6)
Lymph node positive, *n* (%)	7 (0.4)
Positive margins, *n* (%)	39 (14)
BCR, *n* (%)	37 (16)
Time to BCR, median (range), years	2 (1–10)
Follow-up, median (range), years	4 (1–14)
Molecular parameters (measured in biopsy)	
PTEN and *MYC* status, *n* (%)	
PTEN intact *MYC* intact	96 (53)
PTEN intact *MYC* gain	35 (19.3)
PTEN loss *MYC* intact	33 (18.3)
PTEN loss *MYC* gain	17 (9.3)

BCR, biochemical recurrence; PSA, prostate-specific antigen; RP, radical prostatectomy.

In total, 188 of the 277 cases (68%) had evaluable *MYC* status at biopsy, whereas 248 cases (90%) had evaluable PTEN status and 181 cases (65%) had evaluable *MYC* and PTEN status (Figure 1). Of the latter group, 19% had *MYC* gain with intact PTEN, 18% had PTEN loss without *MYC* gain, and 9% had *MYC* gain and PTEN loss. At radical prostatectomy, most patients with up-grading had Grade Group 3 tumor present (14%), whereas 27% of cases had extraprostatic extension, 4% of cases had seminal vesicle involvement, and 0.4% of cases had lymph node involvement. Although 14% of cases had positive surgical margins, these were categorized as organ confined unless there was independent evidence of extraprostatic extension. In total, 16% had subsequent biochemical recurrence.

### Association of Morphologic and Molecular Findings with Preoperative Clinicopathologic Parameters

The preoperative clinicopathologic parameters stratified by morphologic and molecular findings were examined next (Table 2). Patients with intraductal spread of carcinoma in the biopsy tended to be older (*P* = 0.03) and have a higher maximum percentage of tumor involvement on a biopsy core (*P* = 0.011) and a higher percentage of Gleason pattern 4 carcinoma (*P* = 0.007). Presence of intraductal carcinoma was not significantly associated with patient race, preoperative PSA level, clinical stage, fraction of biopsy cores involved by cancer, or presence of bilateral disease on biopsy. Patients with large cribriform Gleason pattern 4 carcinoma present (maximal diameter >200 μm) in biopsy cores had a higher clinical stage (*P* = 0.02), higher maximum percentage of tumor involvement on any biopsy core (*P* = 0.001), and higher percentage of Gleason pattern 4 carcinoma (*P* < 0.0001). Cribriform carcinoma at biopsy (maximal diameter >200 μm) was not significantly associated with patient age, race, preoperative PSA level, fraction of biopsy cores involved by cancer, or presence of bilateral disease on biopsy.

**Table 2:** Clinicopathologic Variables Stratified by Intraductal and Cribriform Carcinoma Status or PTEN/*MYC* Status

Variable	Intraductal Carcinoma			Cribriform Gleason Pattern 4 (>200 μm)			*MYC* and PTEN Dual Status				*P*
	Positive	Negative	*P*	Positive	Negative	*P*	*MYC* Intact PTEN Intact	*MYC* Gain PTEN Intact	*MYC* Intact PTEN Loss	*MYC* Gain PTEN Loss	
Preoperative parameters											
Age, median (range), years	65 (48–72)	62 (41–75)	0.03	64 (45–72)	62 (41–75)	0.09	62.5 (41–71)	63 (45–72)	63 (41–71)	63 (47–74)	0.98
Self-reported African American race, *n* (%)	3 (9.6)	44 (19)	0.28	5 (9.6)	42 (20)	0.11	17 (19)	8 (24)	2 (6)	2 (14)	0.26
PSA, median (range)	4.85 (1.5–23.4)	5.5 (1.3–33.8)	0.23	5.5 (1.5–30.3)	5.27 (1.3–33.8)	0.24	5.36 (1.30–33.80)	5.9 (1.50–16.79)	5 (1.30–30.30)	4.74 (3.40–24.90)	0.55
Clinical stage, *n* (%)											
T1c	17 (56)	177 (75)	0.15	30 (57.6)	164 (76)	0.02	68 (72)	25 (75)	19 (65)	8 (50)	0.26
T2a	5 (16)	27 (12)		12 (23)	21 (10)		12 (12)	6 (18)	5 (17)	4 (25)	
≥T2b	8 (26)	31 (13)		10 (19.4)	29 (14)		14 (14)	2 (6)	5 (17)	4 (25)	
Maximum percentage of tumor involvement, median (range)	70 (15–100)	60 (5–100)	0.011	70 (15–100)	60 (5–100)	0.001	60 (5–100)	70 (20–100)	70 (15–100)	90 (35–100)	0.0007
Fraction cores involved, median (range)	0.33 (0.06–0.91)	0.3 (0.05–1)	0.93	0.35 (0.05–0.92)	0.3 (0.05–1)	0.24	0.28 (0.05–1)	0.33 (0.15–1)	0.33 (0.07–0.92)	0.4 (0.18–0.91)	0.29
Bilateral disease, *n* (%)	17 (54)	131 (53)	1	29 (52)	119 (53)	1	51 (53)	19 (54)	20 (60)	10 (58)	0.88
Gleason pattern 4, median (range), %	20 (1–90)	10 (0–90)	0.007	30 (1–90)	10 (0–90)	<0.0001	10 (0–90)	20 (1–90)	20 (1–50)	30 (10–70)	0.07
Postoperative parameters											
Grade Group at RP, *n* (%)											
1 (Gleason score 6)	8 (25.8)	70 (28.6)	<0.0001	6 (15.8)	33 (27.7)	<0.0001	30 (31)	8 (23)	7 (22)	1 (6)	0.15
2 (Gleason score 3 + 4 = 7)	8 (25.8)	139 (57)		19 (50)	74 (62)		52 (54)	21 (60)	19 (57)	8 (47)	
3 (Gleason score 4 + 3 = 7)	13 (42)	25 (10)		11 (29)	9 (7.5)		10 (10)	4 (11)	6 (18)	7 (41)	
4 (Gleason score 8)	1 (3.2)	5 (2)		2 (5.2)	1 (0.8)		2 (2)	1 (3)	0	1 (6)	
5 (Gleason score 9–10)	1 (3.2)	5 (2)		0	2 (1.6)		2 (2)	1 (3)	1 (3)	0	
Non–organ-confined disease, *n* (%)	18 (58)	73 (29)	0.003	30 (54)	62 (28)	0.0003	19 (19)	15 (42)	16 (48)	12 (70)	<0.0001
Biochemical recurrence, *n* (%)	10 (38)	27 (13)	0.004	15 (31)	22 (12.7)	0.005	7 (9)	5 (16)	7 (28)	4 (26)	0.07

BCR, biochemical recurrence; PSA, prostate-specific antigen; RP, radical prostatectomy.

Continuous variables (age, PSA, percentage of tumor involvement, and fraction cores positive) were compared using Wilcoxon test for tumors with intraductal carcinoma and cribriform lesions. These same variables were compared by the Kruskal-Wallis test for dual PTEN/*MYC* status. Categorical variables were compared by using the χ^2^ test.

**Supplemental Figure S3:**
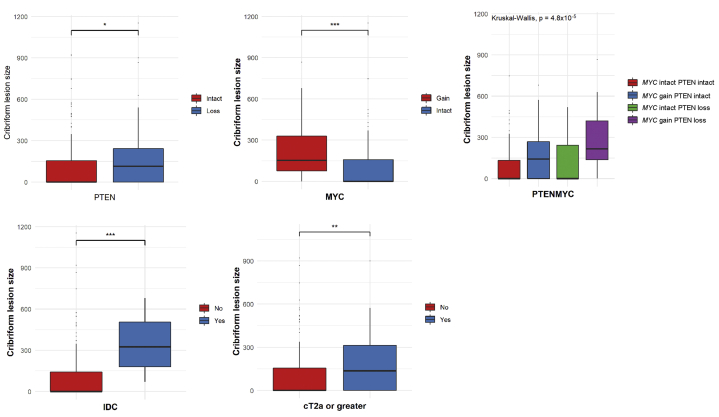
Association of maximal cribriform diameter at biopsy with preoperative and molecular characteristics. ∗*P* < 0.05, ∗∗*P* < 0.01, and ∗∗∗*P* < 0.001. cT2a, clinical stage T2a; IDC, intraductal carcinoma.

Patients with *MYC* gain or PTEN loss or both alterations at biopsy tended to have higher maximum percentage of tumor involvement on any biopsy core (*P* = 0.0007), but molecular findings were not significantly associated with patient age, race, preoperative PSA level, clinical stage, fraction of biopsy cores involved by cancer or presence of bilateral disease on biopsy, or percentage of Gleason pattern 4 carcinoma (Table 2). *MYC* gain and PTEN loss were significantly associated with the presence of intraductal carcinoma at biopsy by logistic regression analyses (OR, 13.33; 95% CI, 3.85–49.67; *P* < 0.0001 for combined *MYC* gain and PTEN loss) (Supplemental Table S3). Cases with *MYC* gain and PTEN loss were also more likely to have cribriform Gleason pattern 4 carcinoma (>200 μm) at biopsy by logistic regression (OR, 8.85; 95% CI, 2.96–27.82; *P* = 0.0001 for combined PTEN loss and *MYC* gain) (Supplemental Table S4). Similarly, when cribriform Gleason pattern 4 carcinoma maximal diameter was examined as a continuous variable, biopsy specimens with *MYC* gain or PTEN loss or both alterations had significantly larger cribriform foci (*P* < 0.0001) than those without these molecular alterations (Supplemental Figure S3).

### Association of Morphologic Findings and Molecular Status with Postoperative Clinicopathologic Parameters

**Supplemental Figure S4:**
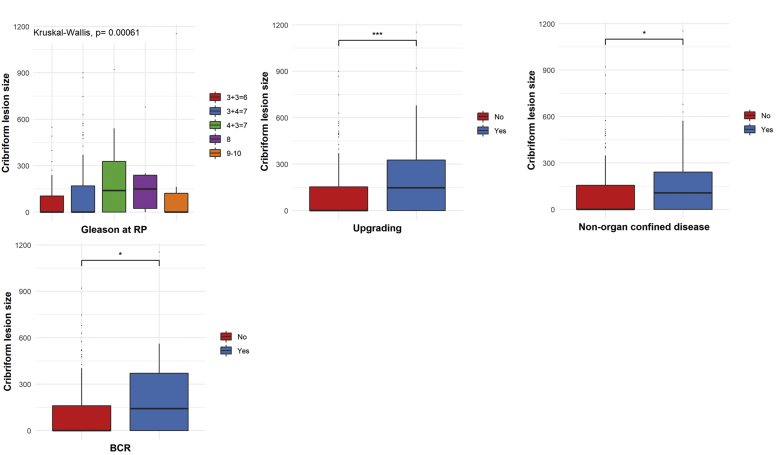
Association of maximal cribriform diameter at biopsy with postoperative characteristics at radical prostatectomy (RP). ∗*P* < 0.05, ∗∗∗*P* < 0.001. BCR, biochemical recurrence.

Among post-operative parameters (Table 2), the presence of intraductal carcinoma at biopsy was significantly associated with increasing Grade Group at radical prostatectomy (*P* < 0.0001) and non–organ-confined disease (defined as extraprostatic extension, seminal vesicle invasion, and/or lymph node involvement; *P* = 0.003) at radical prostatectomy. Similarly, the presence of cribriform Gleason pattern 4 carcinoma at biopsy (maximal diameter >200 μm) was significantly associated with increasing Grade Group (*P* < 0.0001) and non–organ-confined disease (*P* = 0.0003) at radical prostatectomy. When the maximal diameter of cribriform Gleason pattern 4 carcinoma was examined as a continuous variable, Grade Group at radical prostatectomy was significantly associated with biopsy cribriform size (*P* = 0.0006) (Supplemental Figure S4). Joint *MYC*/PTEN status at biopsy was not significantly associated with Grade Group at radical prostatectomy. However, *MYC*/PTEN status was linked to a higher risk of non–organ-confined disease, in which 70% of tumors with both *MYC* gain and PTEN loss at biopsy had this pathologic feature at radical prostatectomy compared with only 19% of tumors without *MYC* gain or PTEN loss (*P* < 0.0001) (Table 2).

### Univariable and Multivariable Analysis of Association of PTEN-*MYC* Status with Adverse Pathologic Features at Radical Prostatectomy

Up-grading and non–organ-confined disease are adverse pathologic features at radical prostatectomy. Since up-grading in our cohort was largely limited to Grade Group 3 (Table 1), the adverse outcome of non–organ-confined disease at surgery was studied. On univariable logistic regression analysis, as reported previously,^17^ biopsy tumor volume metrics were significantly associated with the risk of non–organ-confined disease (Table 3). The presence of large cribriform Gleason pattern 4 carcinoma (maximal diameter >200 μm) (OR, 3.01; 95% CI, 1.59–5.77; *P* = 0.0007) and intraductal spread of carcinoma (OR, 3.03; 95% CI, 1.42-6.61; *P* = 0.0043) at biopsy were both associated with a significantly higher OR for non–organ-confined disease at radical prostatectomy (Table 3). In contrast, cribriform lesions ≤200 μm and percentage of Gleason pattern 4 carcinoma were not significantly associated with non–organ-confined disease (Table 3). On univariable analysis, *MYC*/PTEN status at biopsy was also significantly associated with non–organ-confined disease, with cases showing *MYC* gain and PTEN loss having the highest OR for non–organ-confined disease compared with cases without either alteration, although because of the low number of cases the OR estimates were somewhat unstable for this group (OR, 10.01; 95% CI, 3.36–34.34; *P* < 0.0001). In a multivariable model that included all parameters significant on univariable analyses at biopsy, only *MYC*-PTEN status remained significantly associated with non–organ-confined disease at radical prostatectomy, with *MYC* gain (OR, 3.02; 95% CI, 1.19–7.77; *P* = 0.01) and PTEN loss (OR, 2.76; 95% CI, 1.10–6.98; *P* = 0.02) individually significantly associated with non–organ-confined disease and dual alterations (*MYC* gain with PTEN loss) showing the strongest association (OR, 6.23; 95% CI, 1.74–24.55; *P* = 0.005) (Table 3).

**Table 3:** Univariable and Multivariable Logistic Regression Results for Non–Organ-Confined Disease (Extraprostatic Extension, Seminal Vesicle Invasion, and/or Lymph Node Involvement) at Radical Prostatectomy

Variable	Univariable		Multivariable	
	*P*	OR (95% CI)	*P*	OR (95% CI)
African American ancestry	0.07	0.52 (0.24–1.03)	—	—
Age	0.13	1.02 (0.99–1.06)	—	—
PSA	0.98	1.00 (0.99–1.00)	—	—
Percentage of tumor involvement	0.0008∗	2.73 (1.53–5.05)	0.20	1.84 (0.73–4.90)
Fraction cores involved	0.0006∗	7.74 (2.41–25.92)	0.12	4.12 (0.69–25.45)
cT2a or greater	0.05	1.78 (0.98–3.22)	—	—
Cribriform Gleason pattern 4 carcinoma (reference: no cribriform)				
≤200 um	0.85	0.94 (0.50–1.73)	0.07	0.42 (0.15–1.05)
>200 um	0.0007∗	3.01 (1.59–5.77)	0.51	1.38 (0.51–3.65)
Intraductal carcinoma	0.0043∗	3.03 (1.42–6.61)	0.09	2.70 (0.84–8.95)
Percentage of Gleason pattern 4	0.93	0.99 (0.98–1.01)	—	—
PTEN loss	<0.0001∗	2.90 (1.86–6.04)	—	—
*MYC* gain	0.0005∗	3.09 (1.63–5.92)	—	—
*MYC* and PTEN (reference: *MYC* intact PTEN intact)				
*MYC* gain PTEN intact	0.007∗	3.08 (1.35–7.05)	0.01∗	3.02 (1.19–7.77)
*MYC* intact PTEN loss	0.003∗	3.42 (1.48–7.95)	0.02∗	2.76 (1.10–6.98)
*MYC* gain PTEN loss	<0.0001∗	10.01 (3.36–34.34)	0.005∗	6.23 (1.74–24.55)

OR, odds ratio; PSA, prostate-specific antigen.

∗ Variables with any categories showing *P* < 0.05 in the univariable model were included in the multivariable analysis.

To determine whether *MYC* or PTEN status added information to preoperative variables commonly used in prostate cancer risk assessment algorithms, the ROC curves were assessed for the presence of non–organ-confined disease at radical prostatectomy. In a baseline regression model that included age, PSA, clinical stage, and proportion of biopsy cores positive for tumor (Grade Group was excluded because all biopsy specimens in this cohort were Grade Group 2), the AUC was 0.68. Addition of presence or absence of cribriform or intraductal carcinoma at biopsy marginally improved the AUC to 0.69, whereas further addition of percentage of Gleason pattern 4 carcinoma to this model did not appreciably change the AUC (0.69). Of interest, addition of joint *MYC* and PTEN status at biopsy to the baseline model that included age, PSA, clinical stage, proportion of positive cores, presence of cribriform or intraductal carcinoma, and percentage of Gleason pattern 4 carcinoma further increased the AUC to 0.75. Taken together, these data suggest there is added information provided by the *MYC*-PTEN molecular status to baseline clinicopathologic characteristics.

### Association of *MYC*/PTEN Status with Biochemical Recurrence after Radical Prostatectomy

**Figure 2:**
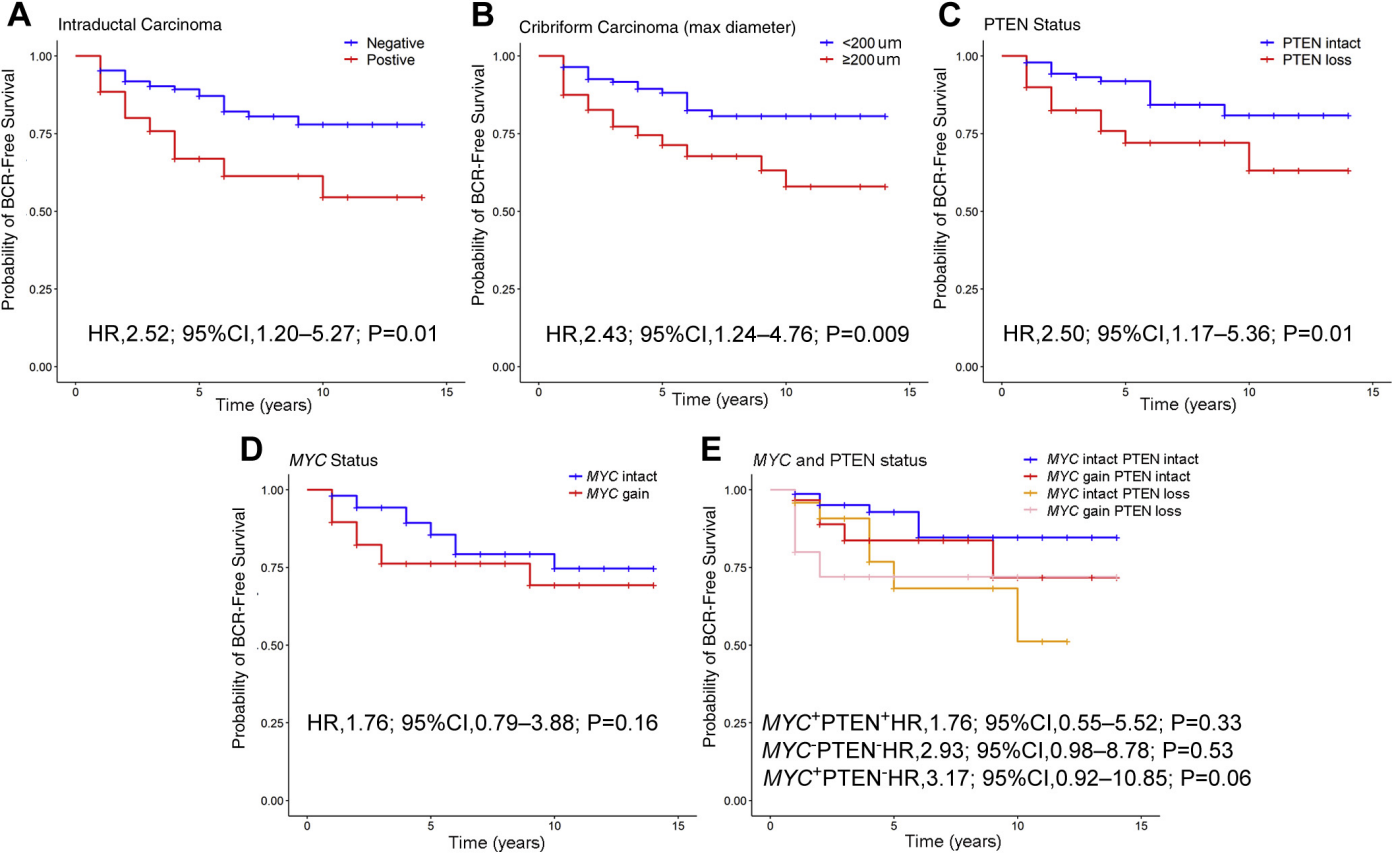
Kaplan-Meier curves for probability of biochemical recurrence stratified by pathologic and molecular parameters. Hazard ratios for Cox proportional hazards regression models are shown on each panel. **A:** Stratification by presence or absence of intraductal carcinoma. **B:** Stratification by presence or absence of cribriform Gleason pattern 4 carcinoma (maximum diameter >200 μm). **C:** Stratification by presence or absence of PTEN loss by gene-protein assay. **D:** Stratification by presence or absence of *MYC* amplification by gene-protein assay. **E:** Stratification by combined *MYC*-PTEN status.

Finally, the association of morphologic and molecular parameters was examined with PSA recurrence after radical prostatectomy. Overall, 37 of 220 patients (16%) had PSA recurrence after radical prostatectomy in the cohort with a median follow-up time of 4 years (interquartile range, 1 to 14 years). By log-rank analysis, the presence of intraductal carcinoma at biopsy (*P* = 0.012), cribriform Gleason pattern 4 carcinoma at biopsy (*P* = 0.007), and PTEN loss at biopsy (*P* = 0.014) were significantly associated with decreased time to recurrence (Figure 2A-C). Although *MYC* gain and dual *MYC*/PTEN status did not reach statistical significance, there was a trend toward more rapid recurrence for patients with these alterations at biopsy (Figure 2D-E).

## Discussion

Spread to extraprostatic soft tissues or seminal vesicles and pelvic lymph nodes (non–organ-confined disease) is a critical indicator of potentially aggressive prostate cancer and is considered an adverse pathologic feature at radical prostatectomy. Key clinical decisions for patients undergoing radical prostatectomy, such as performing nerve sparing surgery^22^ and the need for pelvic lymphadenectomy,^23^ hinge on the magnitude of this risk. Furthermore, in patients with a very high baseline risk of non–organ-confined disease, radiation and hormonal therapy may be preferred over surgical treatment. Prostate needle core biopsies^24^ or multiparametric magnetic resonance imaging studies^25^ are relatively specific for the presence of non–organ-confined disease; however, these techniques are insensitive for this feature. Given that treatment planning involves shared decision-making between the patient and the clinician, having additional data available to better understand the likelihood of adverse patient outcomes, such as non–organ-confined disease, would facilitate this process for both parties.^26^

Molecular biomarkers may provide useful information to guide clinical decision-making in Grade Group 2 prostate cancer. RNA-based prognostic tests have clinical utility in some settings but are not in widespread use because of prohibitive costs, the need for a large amount of tissue, and difficulties of RNA preservation in FFPE samples.^7^^,^^8^ The loss of the tumor suppressor *PTEN* is one of the most common alterations found in primary prostate cancer, and it is reproducibly associated with worse prognosis in numerous studies.^10^, ^11^, ^12^, ^13^^,^^27^, ^28^, ^29^, ^30^, ^31^ *MYC* or 8q amplification is another independent predictor of tumor progression, particularly in the setting of Grade Group 2 or 3 cancer.^14^^,^^15^^,^^32^^,^^33^ In a previous study by collaborators at our institution, *MYC* gain assessed by fluorescence *in situ* hybridization was significantly more common among Gleason pattern 3 tumor sampled from Grade Group 2 or Grade Group 3 prostatectomy samples than Gleason pattern 3 tumor sampled from Grade Group 1 tumors.^14^ In older comparative genomic hybridization studies, 8q gain was similarly associated with worse survival among Gleason score 7 prostate cancer.^32^

Taken together, these older studies prompted us to ask whether *MYC* gain could be assessed via chromogenic assays in a dual-plex fashion with an immunohistochemical assay for PTEN. Using technology originally adapted for HER2/neu assessment in breast cancer,^16^ we developed a new clinical grade, automated chromogenic assay to assess *MYC* and PTEN status simultaneously on a single biopsy slide. This assay can be run and interpreted in any Clinical Laboratory Improvement Amendments–accredited immunohistochemistry laboratory on the Ventana BenchMark platform. PTEN status assessment in this dual-plex assay was rigorously validated by comparison with the performance of a genetically validated PTEN immunohistochemistry assay in the same cohort, as well as to another anti-PTEN antibody, with good agreement among the various assays. In a homogeneous cohort of Grade Group 2 biopsy samples from patients who underwent subsequent radical prostatectomy, the AUC for a baseline model using the preoperative variables from standard preoperative risk calculators was found to be increased from 0.68 to 0.69 with the inclusion of intraductal or cribriform carcinoma status and to 0.75 with the inclusion of *MYC*/PTEN status. These data, if validated in other independent cohorts, suggest that simultaneous *MYC*/PTEN status assessment may be useful in the intermediate-risk biopsy setting to identify patients at high risk for non–organ-confined prostate cancer at surgery. This information could be useful to help determine which patients are not candidates for active surveillance or nerve-sparing surgery and to stratify patients who could be at high risk for recurrence.

In addition to molecular markers, refinements to pathologic grading of prostate cancer may add to the prognostic power of Grade Group, particularly for intermediate-risk patients. Multiple studies and meta-analyses have found that the presence of intraductal spread of carcinoma at biopsy adds independent prognostic information beyond that of Grade Group.^2^^,^^3^^,^^5^^,^^34^ Similarly, the presence of invasive cribriform Gleason pattern 4 carcinoma at biopsy may be useful, particularly in Grade Group 2 prostate cancer, for predicting outcomes.^5^^,^^35^, ^36^, ^37^, ^38^ However, to date, the data on cribriform pattern as a marker of worse prognosis have been confounded by lack of consensus on cribriform carcinoma size cutoffs and the ability to distinguish cribriform carcinoma from intraductal spread, which requires immunohistochemical stains in most cases.^4^^,^^37^^,^^39^, ^40^, ^41^, ^42^, ^43^ Although several previous studies have noted that larger cribriform proliferations are associated with worse prognosis,^42^ others have identified a worse prognosis for cribriform lesions independent of size.^37^^,^^39^ This lack of consensus may be attributable to the fact that larger size has been defined variably, from cribriform lesions with >12 lumens^39^ to glandular enlargement more than two-fold that of normal benign glands.^42^ In addition, it is often challenging to distinguish invasive cribriform lesions from intraductal carcinoma without immunohistochemical stains, and many studies have not separated these two entities to evaluate their independent prognostic potential.^3^^,^^38^

To begin to address these knowledge gaps, in the current study, the tumor was digitally measured to precisely define cribriform Gleason pattern 4 lesion size, and p63 immunostaining was used to differentiate intraductal from invasive cribriform Gleason pattern 4. Using AUC analysis, it was found that 200 μm was an optimal cut point to distinguish cribriform Gleason pattern 4 lesions associated with non–organ-confined disease. The presence of large (>200 μm) cribriform architecture but not smaller lesions was significantly associated with increasing higher clinical stage and higher tumor volume preoperatively and associated with higher Grade Group and non–organ-confined disease at radical prostatectomy. Notably, even in the absence of a defined cut point, it was found that cases with *MYC* gain and/or PTEN loss had significantly larger cribriform lesions compared with those with intact *MYC* and PTEN. This finding constitutes some of the first evidence that larger cribriform lesions are molecularly distinct from smaller ones and supports the use of some metric of cribriform size in prognostic algorithms that include cribriform architecture. Defined by the presence of p63-positive basal cells around a solid or densely cribriform intraductal proliferation, the presence of intraductal carcinoma was also associated with adverse preoperative and postoperative features, including higher biopsy tumor volume, as well as increased Grade Group and non–organ-confined disease after surgery.

There are some limitations to the current study. First, to preserve tissue for future potential clinical molecular studies, only one index tumor block for each case was submitted for the dual-plex chromogenic assay, which may inadequately characterize genomically heterogeneous tumors.^44^ Similarly, only one tumor block was evaluated for the presence of cribriform Gleason pattern 4 architecture or intraductal carcinoma. To estimate the extent of error in our classification for the presence of cribriform Gleason pattern 4 architecture or intraductal carcinoma, all additional Grade Group 2 cancer–containing blocks were examined for cases that lacked large cribriform or intraductal carcinoma in the initial analysis. Overall, 15 cases were misclassified as lacking cribriform Gleason pattern 4 architecture (maximal diameter >200 μm) or intraductal carcinoma based on the initial analysis, amounting to 5% of the entire cohort. This finding suggests that the magnitude of error introduced by our sampling methods may be relatively small, although it still remains a limitation. An additional limitation is that there was a relatively high rate of unevaluable samples, particularly for the *MYC* assay, because of the use of archival paraffin material. Despite these shortcomings, both molecular markers and refined histologic classification remained significantly associated with outcomes in multivariable models. Finally, there was a relatively low number of biochemical recurrence events because the cases studied were exclusively Grade Group 2 at biopsy and all underwent definitive surgical therapy. Thus, multivariable models of biochemical recurrence were not constructed, and the outcome of non–organ-confined disease was examined as a surrogate marker. However, because the presence or absence of non–organ-confined disease can help to guide surgical planning, this outcome is useful on its own as well.

In conclusion, this study indicates that a clinical grade, multiplex gene-protein panel can be developed for adverse genomic alterations in primary prostate cancer and may add to contemporary clinicopathologic parameters in identifying patients at increased risk of non–organ-confined disease before surgery. It was also found that contemporary adverse pathological features, such as cribriform Gleason pattern 4 carcinoma and intraductal carcinoma, are associated with underlying molecular alterations, including *MYC* and PTEN genomic status, providing additional support for their use in pathologic evaluation. Future studies will prospectively evaluate the clinical utility of joint *MYC*/PTEN status assessment in the setting of intermediate-risk prostate cancer.

## References

[1] Musunuru H.B., Yamamoto T., Klotz L., Ghanem G., Mamedov A., Sethukavalan P., Jethava V., Jain S., Zhang L., Vesprini D., Loblaw A. (2016). Active surveillance for intermediate risk prostate cancer: survival outcomes in the sunnybrook experience. J Urol.

[2] Epstein J.I., Amin M.B., Fine S.W., Algaba F., Aron M., Baydar D.E. (2020). The 2019 Genitourinary Pathology Society (GUPS) White Paper on Contemporary Grading of Prostate Cancer. Arch Pathol Lab Med.

[3] Kweldam C.F., Kummerlin I.P., Nieboer D., Verhoef E.I., Steyerberg E.W., van der Kwast T.H., Roobol M.J., van Leenders G.J. (2016). Disease-specific survival of patients with invasive cribriform and intraductal prostate cancer at diagnostic biopsy. Mod Pathol.

[4] Hollemans E., Verhoef E.I., Bangma C.H., Rietbergen J., Osanto S., Pelger R.C.M., van Wezel T., van der Poel H., Bekers E., Helleman J., Roobol M.J., van Leenders G. (2020). Cribriform architecture in radical prostatectomies predicts oncological outcome in Gleason score 8 prostate cancer patients. Mod Pathol.

[5] van Leenders G., Kweldam C.F., Hollemans E., Kummerlin I.P., Nieboer D., Verhoef E.I., Remmers S., Incrocci L., Bangma C.H., van der Kwast T.H., Roobol M.J. (2020). Improved prostate cancer biopsy grading by incorporation of invasive cribriform and intraductal carcinoma in the 2014 Grade Groups. Eur Urol.

[6] Kato M., Tsuzuki T., Kimura K., Hirakawa A., Kinoshita F., Sassa N., Ishida R., Fukatsu A., Kimura T., Funahashi Y., Matsukawa Y., Hattori R., Gotoh M. (2016). The presence of intraductal carcinoma of the prostate in needle biopsy is a significant prognostic factor for prostate cancer patients with distant metastasis at initial presentation. Mod Pathol.

[7] Lotan T.L., Tomlins S.A., Bismar T.A., Van der Kwast T.H., Grignon D., Egevad L., Kristiansen G., Pritchard C.C., Rubin M.A., Bubendorf L. (2020). Report from the International Society of Urological Pathology (ISUP) Consultation Conference on Molecular Pathology of Urogenital Cancers, I: molecular biomarkers in prostate cancer. Am J Surg Pathol.

[8] Ross A.E., D'Amico A.V., Freedland S.J. (2016). Which, when and why? rational use of tissue-based molecular testing in localized prostate cancer. Prostate Cancer Prostatic Dis.

[9] Lobo J.M., Trifiletti D.M., Sturz V.N., Dicker A.P., Buerki C., Davicioni E., Cooperberg M.R., Karnes R.J., Jenkins R.B., Den R.B., Showalter T.N. (2017). Cost-effectiveness of the Decipher genomic classifier to guide individualized decisions for early radiation therapy after prostatectomy for prostate cancer. Clin Genitourinary Cancer.

[10] Lotan T.L., Gurel B., Sutcliffe S., Esopi D., Liu W., Xu J., Hicks J.L., Park B.H., Humphreys E., Partin A.W., Han M., Netto G.J., Isaacs W.B., De Marzo A.M. (2011). PTEN protein loss by immunostaining: analytic validation and prognostic indicator for a high risk surgical cohort of prostate cancer patients. Clin Cancer Res.

[11] Ahearn T.U., Pettersson A., Ebot E.M., Gerke T., Graff R.E., Morais C.L., Hicks J.L., Wilson K.M., Rider J.R., Sesso H.D., Fiorentino M., Flavin R., Finn S., Giovannucci E.L., Loda M., Stampfer M.J., De Marzo A.M., Mucci L.A., Lotan T.L. (2016). A prospective investigation of PTEN loss and ERG expression in lethal prostate cancer. J Natl Cancer Inst.

[12] Lotan T.L. (2016). Analytic validation of a clinical-grade PTEN immunohistochemistry assay in prostate cancer by comparison with PTEN FISH. Hum Pathol.

[13] Jamaspishvili T., Berman D.M., Ross A.E., Scher H.I., De Marzo A.M., Squire J.A., Lotan T.L. (2018). Clinical implications of PTEN loss in prostate cancer. Nat Reviews Urol.

[14] Trock B.J., Fedor H., Gurel B., Jenkins R.B., Knudsen B.S., Fine S.W., Said J.W., Carter H.B., Lotan T.L., De Marzo A.M. (2016). PTEN loss and chromosome 8 alterations in Gleason grade 3 prostate cancer cores predicts the presence of un-sampled grade 4 tumor: implications for active surveillance. Mod Pathol.

[15] Fromont G., Godet J., Peyret A., Irani J., Celhay O., Rozet F., Cathelineau X., Cussenot O. (2013). 8q24 amplification is associated with Myc expression and prostate cancer progression and is an independent predictor of recurrence after radical prostatectomy. Hum Pathol.

[16] Nitta H., Kelly B.D., Padilla M., Wick N., Brunhoeber P., Bai I., Singh S., Ranger-Moore J., Bieniarz C., Tsuda H., Grogan T.M. (2012). A gene-protein assay for human epidermal growth factor receptor 2 (HER2): brightfield tricolor visualization of HER2 protein, the HER2 gene, and chromosome 17 centromere (CEN17) in formalin-fixed, paraffin-embedded breast cancer tissue sections. Diagn Pathol.

[17] Guedes L.B., Tosoian J.J., Hicks J., Ross A.E., Lotan T.L. (2017). PTEN loss in Gleason score 3 + 4 = 7 prostate biopsies is associated with nonorgan confined disease at radical prostatectomy. J Urol.

[18] Karram S., Trock B.J., Netto G.J., Epstein J.I. (2011). Should intervening benign tissue be included in the measurement of discontinuous foci of cancer on prostate needle biopsy? correlation with radical prostatectomy findings. Am J Surg Pathol.

[19] Epstein J.I., Allsbrook W.C., Amin M.B., Egevad L.L., Committee I.G. (2005). The 2005 International Society of Urological Pathology (ISUP) Consensus Conference on Gleason Grading of Prostatic Carcinoma. Am J Surg Pathol.

[20] Lotan T.L., Wei W., Morais C.L., Hawley S.T., Fazli L., Hurtado-Coll A., Troyer D., McKenney J.K., Simko J., Carroll P.R., Gleave M., Lance R., Lin D.W., Nelson P.S., Thompson I.M., True L.D., Feng Z., Brooks J.D. (2016). PTEN loss as determined by clinical-grade immunohistochemistry assay is associated with worse recurrence-free survival in prostate cancer. Eur Urol Focus.

[21] Stasik C.J., Nitta H., Zhang W., Mosher C.H., Cook J.R., Tubbs R.R., Unger J.M., Brooks T.A., Persky D.O., Wilkinson S.T., Grogan T.M., Rimsza L.M. (2010). Increased MYC gene copy number correlates with increased mRNA levels in diffuse large B-cell lymphoma. Haematologica.

[22] Martini A., Cumarasamy S., Haines K.G., Tewari A.K. (2019). An updated approach to incremental nerve sparing for robot-assisted radical prostatectomy. BJU Int.

[23] Briganti A., Larcher A., Abdollah F., Capitanio U., Gallina A., Suardi N., Bianchi M., Sun M., Freschi M., Salonia A., Karakiewicz P.I., Rigatti P., Montorsi F. (2012). Updated nomogram predicting lymph node invasion in patients with prostate cancer undergoing extended pelvic lymph node dissection: the essential importance of percentage of positive cores. Eur Urol.

[24] Faisal F.A., Tosoian J.J., Han M., Macura K.J., Pavlovich C.P., Lotan T.L. (2019). Clinical, pathological and oncologic findings of radical prostatectomy with extraprostatic extension diagnosed on preoperative prostate biopsy. J Urol.

[25] de Rooij M., Hamoen E.H., Witjes J.A., Barentsz J.O., Rovers M.M. (2016). Accuracy of magnetic resonance imaging for local staging of prostate cancer: a diagnostic meta-analysis. Eur Urol.

[26] Sanda M.G., Cadeddu J.A., Kirkby E., Chen R.C., Crispino T., Fontanarosa J., Freedland S.J., Greene K., Klotz L.H., Makarov D.V., Nelson J.B., Rodrigues G., Sandler H.M., Taplin M.E., Treadwell J.R. (2018). Clinically localized prostate cancer: AUA/ASTRO/SUO guideline, part I: risk stratification, shared decision making, and care options. J Urol.

[27] Mehra R., Salami S.S., Lonigro R., Bhalla R., Siddiqui J., Cao X., Spratt D.E., Palapattu G.S., Palanisamy N., Wei J.T., Chinnaiyan A.M., Tomlins S.A. (2018). Association of ERG/PTEN status with biochemical recurrence after radical prostatectomy for clinically localized prostate cancer. Med Oncol.

[28] Bismar T.A., Yoshimoto M., Vollmer R.T., Duan Q., Firszt M., Corcos J., Squire J.A. (2011). PTEN genomic deletion is an early event associated with ERG gene rearrangements in prostate cancer. BJU Int.

[29] Yoshimoto M., Cunha I.W., Coudry R.A., Fonseca F.P., Torres C.H., Soares F.A., Squire J.A. (2007). FISH analysis of 107 prostate cancers shows that PTEN genomic deletion is associated with poor clinical outcome. Br J Cancer.

[30] Leapman M.S., Nguyen H.G., Cowan J.E., Xue L., Stohr B., Simko J., Cooperberg M.R., Carroll P.R. (2018). Comparing prognostic utility of a single-marker immunohistochemistry approach with commercial gene expression profiling following radical prostatectomy. Eur Urol.

[31] Reid A.H., Attard G., Ambroisine L., Fisher G., Kovacs G., Brewer D., Clark J., Flohr P., Edwards S., Berney D.M., Foster C.S., Fletcher A., Gerald W.L., Moller H., Reuter V.E., Scardino P.T., Cuzick J., de Bono J.S., Cooper C.S., Transatlantic Prostate Group (2010). Molecular characterisation of ERG, ETV1 and PTEN gene loci identifies patients at low and high risk of death from prostate cancer. Br J Cancer.

[32] Ribeiro F.R., Jeronimo C., Henrique R., Fonseca D., Oliveira J., Lothe R.A., Teixeira M.R. (2006). 8q gain is an independent predictor of poor survival in diagnostic needle biopsies from prostate cancer suspects. Clin Cancer Res.

[33] Zafarana G., Ishkanian A.S., Malloff C.A., Locke J.A., Sykes J., Thoms J., Lam W.L., Squire J.A., Yoshimoto M., Ramnarine V.R., Meng A., Ahmed O., Jurisica I., Milosevic M., Pintilie M., van der Kwast T., Bristow R.G. (2012). Copy number alterations of c-MYC and PTEN are prognostic factors for relapse after prostate cancer radiotherapy. Cancer.

[34] Miura N., Mori K., Mostafaei H., Quhal F., Motlagh R.S., Pradere B., Laukhtina E., D’Andrea D., Saika T., Shariat S.F. (2020). The prognostic impact of intraductal carcinoma of the prostate: a systematic review and meta-analysis. J Urol.

[35] Kweldam C.F., Kummerlin I.P., Nieboer D., Steyerberg E.W., Bangma C.H., Incrocci L., van der Kwast T.H., Roobol M.J., van Leenders G.J. (2017). Presence of invasive cribriform or intraductal growth at biopsy outperforms percentage grade 4 in predicting outcome of Gleason score 3+4=7 prostate cancer. Mod Pathol.

[36] Tom M.C., Nguyen J.K., Luciano R., Mian O.Y., Stephans K.L., Ciezki J.P., Smile T.D., Wei W., McKenney J.K., Magi-Galluzzi C., Tendulkar R.D. (2019). Impact of cribriform pattern and intraductal carcinoma on gleason 7 prostate cancer treated with external beam radiotherapy. J Urol.

[37] Keefe D.T., Schieda N., El Hallani S., Breau R.H., Morash C., Robertson S.J., Mai K.T., Belanger E.C., Flood T.A. (2015). Cribriform morphology predicts upstaging after radical prostatectomy in patients with Gleason score 3 + 4 = 7 prostate cancer at transrectal ultrasound (TRUS)-guided needle biopsy. Virchows Arch.

[38] Verhoef E.I., Kweldam C.F., Kummerlin I.P., Nieboer D., Bangma C.H., Incrocci L., van der Kwast T.H., Roobol M.J., van Leenders G.J. (2018). Characteristics and outcome of prostate cancer patients with overall biopsy Gleason score 3 + 4 = 7 and highest Gleason score 3 + 4 = 7 or > 3 + 4 = 7. Histopathology.

[39] Iczkowski K.A., Torkko K.C., Kotnis G.R., Wilson R.S., Huang W., Wheeler T.M., Abeyta A.M., La Rosa F.G., Cook S., Werahera P.N., Lucia M.S. (2011). Digital quantification of five high-grade prostate cancer patterns, including the cribriform pattern, and their association with adverse outcome. Am J Clin Pathol.

[40] McKenney J.K., Wei W., Hawley S., Auman H., Newcomb L.F., Boyer H.D., Fazli L., Simko J., Hurtado-Coll A., Troyer D.A., Tretiakova M.S., Vakar-Lopez F., Carroll P.R., Cooperberg M.R., Gleave M.E., Lance R.S., Lin D.W., Nelson P.S., Thompson I.M., True L.D., Feng Z., Brooks J.D. (2016). Histologic grading of prostatic adenocarcinoma can be further optimized: analysis of the relative prognostic strength of individual architectural patterns in 1275 patients from the canary retrospective cohort. Am J Surg Pathol.

[41] Trudel D., Downes M.R., Sykes J., Kron K.J., Trachtenberg J., van der Kwast T.H. (2014). Prognostic impact of intraductal carcinoma and large cribriform carcinoma architecture after prostatectomy in a contemporary cohort. Eur Journal Cancer.

[42] Hollemans E., Verhoef E.I., Bangma C.H., Rietbergen J., Helleman J., Roobol M.J., van Leenders G. (2019). Large cribriform growth pattern identifies ISUP grade 2 prostate cancer at high risk for recurrence and metastasis. Mod Pathol.

[43] Greenland N.Y., Cowan J.E., Zhang L., Carroll P.R., Chan E., Stohr B.A., Simko J.P. (2020). Expansile cribriform Gleason pattern 4 has histopathologic and molecular features of aggressiveness and greater risk of biochemical failure compared to glomerulation Gleason pattern 4. Prostate.

[44] Zheng S.L., Sun J., Cheng Y., Li G., Hsu F.C., Zhu Y., Chang B.L., Liu W., Kim J.W., Turner A.R., Gielzak M., Yan G., Isaacs S.D., Wiley K.E., Sauvageot J., Chen H.S., Gurganus R., Mangold L.A., Trock B.J., Gronberg H., Duggan D., Carpten J.D., Partin A.W., Walsh P.C., Xu J., Isaacs W.B. (2007). Association between two unlinked loci at 8q24 and prostate cancer risk among European Americans. J Natl Cancer Inst.

