# Supplementary material for: Assessment of MYC/PTEN Status by Gene-Protein Assay in Grade Group 2 Prostate Biopsies
Source: J Mol Diagn. 2021 Aug;23(8):1030–41. doi: 10.1016/j.jmoldx.2021.05.006 (PMC8491088; doi:10.1016/j.jmoldx.2021.05.006)
Supplement: Supplemental Table S1 [file mmc1.docx]

**Supplementary Table S1. Concordance between original single-plex assay IHC for PTEN versus dual-plex *MYC-*PTEN assay.** Assays were performed on different levels from the same tumor block. Cohen’s kappa was calculated without missing values and was equal to 0.61. When only intact and loss categories are included, Cohen’s kappa increased to 0.66.

|  |  | | **Dual-plex PTEN score** | | |
| --- | --- | --- | --- | --- | --- |
| **PTEN IHC score** | **Missing** | **Intact** | | **Heterogeneous loss** | **Homogeneous loss** |
| **Missing** | 7 | 10 | | 1 | 5 |
| **Intact** | 12 | 158 | | 4 | 12 |
| **Heterogeneous loss** | 5 | 12 | | 22 | 3 |
| **Homogeneous loss** | 5 | 2 | | 2 | 17 |
