# Supplementary material for: Assessment of MYC/PTEN Status by Gene-Protein Assay in Grade Group 2 Prostate Biopsies
Source: J Mol Diagn. 2021 Aug;23(8):1030–41. doi: 10.1016/j.jmoldx.2021.05.006 (PMC8491088; doi:10.1016/j.jmoldx.2021.05.006)
Supplement: Supplemental Table S2 [file mmc2.docx]

**Supplementary Table S2. Frequency of Intraductal and large (> 200 µm) cribriform Gleason pattern 4 lesions. Chi-square P-value was <0.0001 for this comparison.**

|  | **Large (> 200 µm) cribriform lesion** | |
| --- | --- | --- |
| **Intraductal** | **No** | **Yes** |
| **No** | 213 | 32 |
| **Yes** | 9 | 22 |
