# Supplementary material for: Assessment of MYC/PTEN Status by Gene-Protein Assay in Grade Group 2 Prostate Biopsies
Source: J Mol Diagn. 2021 Aug;23(8):1030–41. doi: 10.1016/j.jmoldx.2021.05.006 (PMC8491088; doi:10.1016/j.jmoldx.2021.05.006)
Supplement: Supplemental Table S3 [file mmc3.docx]

|  | **P** | **OR (95% CI)** |
| --- | --- | --- |
| ***MYC* gain** | 0.02 | 2.54 (1.10-5.88) |
| **PTEN loss** | <0.0001 | 5.01 (2.26-11.47) |
| ***MYC* and PTEN** |  |  |
| *MYC* gain PTEN intact | 0.33 | 1.93 (0.46-7.23) |
| *MYC* intact PTEN loss | 0.007 | 4.80 (1.53-15.83) |
| *MYC* gain PTEN loss | <0.0001 | 13.33 (3.85-49.67) |

**Supplementary Table S3. Association of *MYC* gain and PTEN loss with intraductal carcinoma at biopsy.** The table shows results derived from univariable models. OR = Odds ratio; 95% CI = 95% confidence interval.
