# Supplementary material for: Assessment of MYC/PTEN Status by Gene-Protein Assay in Grade Group 2 Prostate Biopsies
Source: J Mol Diagn. 2021 Aug;23(8):1030–41. doi: 10.1016/j.jmoldx.2021.05.006 (PMC8491088; doi:10.1016/j.jmoldx.2021.05.006)
Supplement: Supplemental Table S4 [file mmc4.docx]

|  | **P** | **OR (95% CI)** |
| --- | --- | --- |
| ***MYC* gain** | 0.0007 | 3.23 (1.63-6.45) |
| **PTEN loss** | 0.001 | 2.73 (1.44-5.12) |
|  |  |  |
| ***MYC* and PTEN** |  |  |
| *MYC* gain PTEN intact | 0.002 | 4.00 (1.61-10.09) |
| *MYC* intact PTEN loss | 0.004 | 3.86 (1.52-9.89) |
| *MYC* gain PTEN loss | 0.0001 | 8.85 (2.96-27.82) |

**Supplementary Table S4. Association of *MYC* gain and PTEN loss with cribriform lesions.** The table shows results derived from univariable models. OR = Odds ratio; 95% CI = 95% confidence interval.
